# L-type association between magnesium intake and human papillomavirus infection in US adult women: based on NHANES 2003–2016 data

**DOI:** 10.3389/fnut.2025.1594489

**Published:** 2025-05-30

**Authors:** Haiwei Chen, Xiaotong Chen, Yuling Chen, Lixin Tang, Wen-Jing Shi, Yu-Hua Ou

**Affiliations:** ^1^Department of Clinical Medicine, The Second Clinical College of Guangzhou Medical University, Guangzhou, China; ^2^Department of Gynecology, The Second Affiliated Hospital of Guangzhou Medical University, Guangzhou Medical University, Guangzhou, China

**Keywords:** HPV infection, magnesium intake, diet management, nutritional regulation, disease prevention

## Abstract

**Background:**

In the post-vaccine era, adjusting living habits and diet structure has become a new way to prevent Human papillomavirus(HPV). Although dietary factors have received much attention, the association of dietary magnesium with HPV infection remains understudied.

**Method:**

Using NHANES cross-sectional data from 2003 to 2016, this study analyzed the relationship between magnesium intake and HPV infection in 7,246 women aged 18–59 years. Weighted logistic regression and subgroup analysis assessed independent links, while curve fitting and threshold analysis defined dose response and saturation.

**Result:**

A significant negative correlation was observed between magnesium intake and the risk of HPV infection. After comprehensive adjustment for potential confounding factors, individuals in the highest quartile of magnesium intake exhibited a statistically significant 29.7% reduction in the risk of HPV infection for each additional unit, compared to those in the lowest quartile (CI:0.554–0.894, *p* = 0.005). Besides, using smooth curve fitting and threshold analysis, we found an L-shaped dose response between magnesium intake and HPV risk. Below 401 mg/day of magnesium, increased intake is inversely correlated with HPV infection risk. Above this threshold, further increases plateaued in risk reduction.

**Conclusion:**

Moderate magnesium intake has a protective effect against HPV infection. Rationally increasing magnesium intake through dietary channels is expected to serve as an effective preventive measure against HPV infection.

## 1 Background

Human papillomavirus (HPV) is a highly prevalent sexually transmitted infection characterized by a high infection rate, with studies estimating that nearly 90% of women will contract HPV at least once in their lifetime (1). Currently, approximately 200 distinct HPV types have been recognized, among which 12 have been classified as high-risk by the International Agency for Research on Cancer (IARC), namely HPV16, 18, 31, 33, 35, 39, 45, 51, 52, 56, 58, and 59 (2). Notably, HPV16 and 18 are significantly associated with a large majority of HPV-related cancer cases (3, 4). Despite the marked decrease in HPV infections and cervical cancer occurrences following the introduction of universal vaccination, the burden of HPV-related diseases remains considerable in developing countries due to limited vaccine accessibility and insufficient vaccination rates (5, 6). Therefore, investigating the potential role of individual lifestyle behaviors and dietary compositions in developing preventive strategies may offer promising new avenues for reducing HPV infection rates.

Magnesium is a pivotal mineral integral to preserving the physiological functions and metabolic equilibrium of human cells. It plays a significant role in various biological processes, such as ion homeostasis, intracellular and extracellular signaling mechanisms (7), the biosynthetic processes of proteins and nucleic acids (8, 9), enzymatic reactions (10), and energy metabolism (11, 12). Dietary magnesium primarily originates from green leafy vegetables, whole grains, nuts, and fish, with an intestinal absorption rate typically ranging from 30 to 50%. The majority of magnesium in the human body is stored in bones (more than 50% of the total), followed by muscles, soft tissues, and organs (about 34 to 39%), while the extracellular fluid contains only 1 to 2% of magnesium (13). Therefore, once the balance of magnesium is disrupted, it will affect the normal biological function and then trigger a series of pathological conditions, which will have adverse effects on the health of the individual.

The HPV virus has a double-stranded DNA structure and replicates itself by forming nuclear plasmids with multiple copies. In this replication cycle, initiation binding protein E2 and DNA replication helicase E1 play a crucial role and are indispensable key protein factors (14). Hence, the life cycle of HPV is closely regulated by the E2 protein. Furthermore, the E2 protein holds a significant position in transcriptional modulation, influencing the expression pattern and spatial organization of the oncogenic genes E6 and E7, and thereby ensuring the stability and persistence of the viral genome (15–17). Studies led by Hannah Lewis and her team have shown that magnesium ions elevate the binding efficiency of the E2 protein and facilitate its transitions among various binding conformations. This effect may stem from magnesium ions modifying the structure and charge arrangement of the E2 protein or DNA, thus promoting the binding interaction (18, 19).

However, current research on the relationship between magnesium intake and HPV infection is still insufficient. Therefore, we used the data resources of the National Health and Nutrition Examination Survey (NHANES) to examine the potential correlation between magnesium intake levels and the presence or absence of HPV infection, in order to provide strong scientific support and practical guidance strategies for the prevention of HPV infection through dietary modification.

## 2 Methods

### 2.1 The data source and the study population

**Figure 1:**
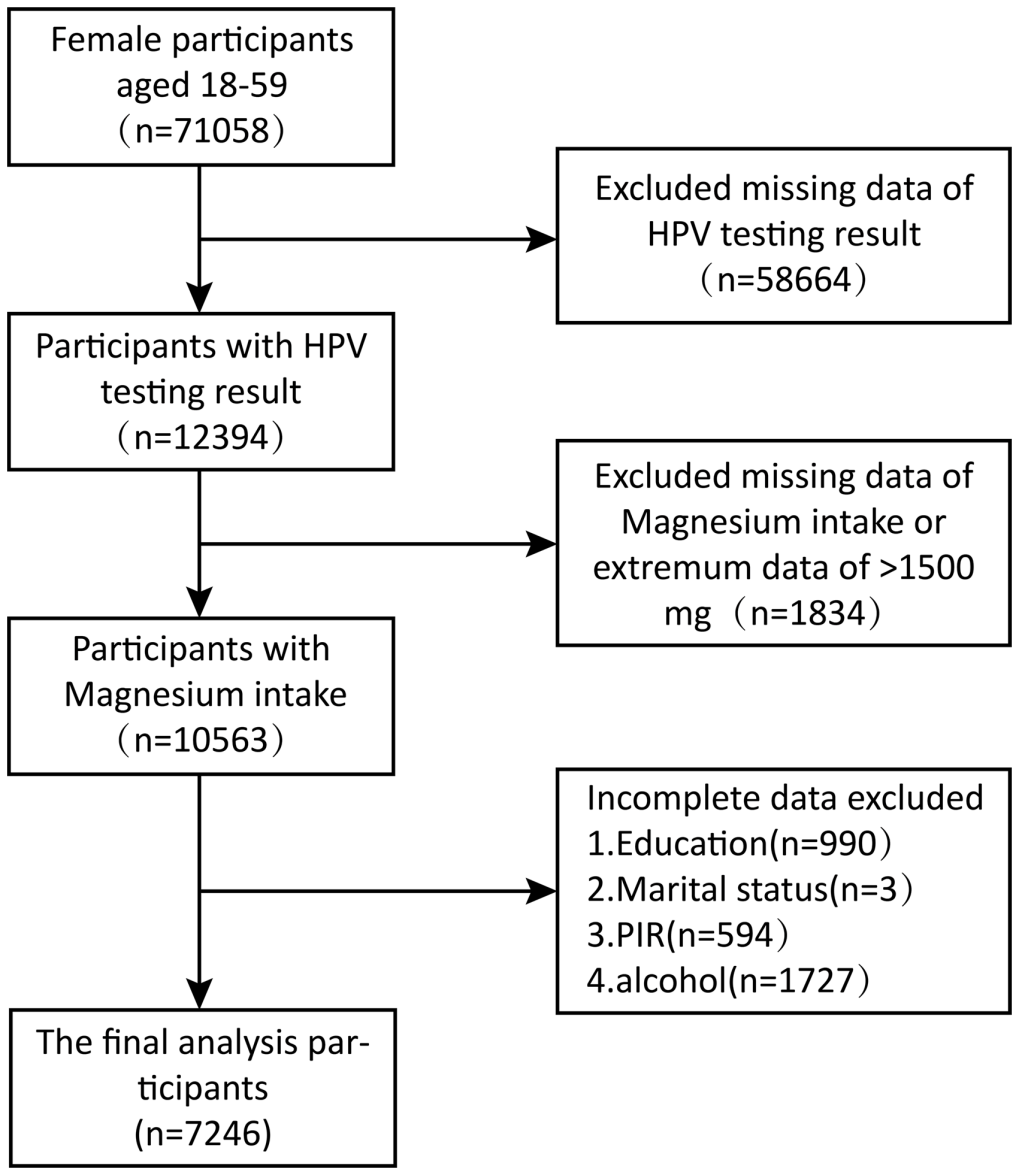
Diagram of NHANES sample selection, 2003–2016.

We executed a cross-sectional study using a sample of seven consecutive data cycles collected by the National Health and Nutrition Examination Survey (NHANES) from 2003 to 2016, including a total of 71,058 participants. And after weighted analysis, it represents a population of 52,022,419. NHANES is a pivotal cross-sectional study conducted by the National Center for Health Statistics (NCHS), an arm of the U.S. Centers for Disease Control and Prevention (CDC). The process of analyzing data in this study was carried out strictly in line with the guidelines and regulatory requirements provided by NHANES. Based on the exhaustive search and strict screening criteria of the NHANES database, 71,058 women aged 18 to 59 from 2003 to 2016 were selected as the base group for this study. In a careful screening process, we excluded 58,664 participants with a lack of HPV infection status information, 1834 with missing or abnormal magnesium intake data (> 1,500 mg), and 3,317 with incomplete data on other key covariates, and finally identified 7,246 eligible participants for inclusion in this study. The sequence of steps in the screening process is clearly shown in Figure 1.

### 2.2 Variables

The core exposure variables of this study were magnesium intake and human papillomavirus (HPV) infection status. The NHANES program assessed food intake through data collection on two different dates: the first face-to-face meal recall interview at a transportable medical examination center (MEC), and the second phone interview three to 10 days after the first interview. In view of the large number of information omissions in the next day’s interviews, we adopted the magnesium intake data from the food intake on the first day of the data analysis and made a scientific estimate of dietary magnesium nutrition according to the Nutritional and Food Database for Diet Studies (FNDDS) published by the United States Department of Agriculture (USDA). In addition, it is crucial to note that the dietary magnesium intake data collected in this study were limited to magnesium directly derived from food and did not take into account magnesium in dietary supplements. For rigorous statistical analysis, we divided the quartile distribution of magnesium intake into the following four statistical intervals: the first quartile interval (Q1), which ranges from 0 mg/day to 173 mg/day; The second quartile range (Q2), ranging from 174 mg/day to 244 mg/day; The third quartile range (Q3), covering 245 mg/day to 332 mg/day; The fourth quartile range (Q4) extends from 333 mg/day to 1,500 mg/day. In the NHANES database, methods for diagnosing human papillomavirus (HPV) infection include collecting cell samples from a participant’s vagina, testing and analyzing DNA extracted from vaginal swabs deploying the Roche prototype linear blotting assay alongside the Roche Linear Array (LA) for genotyping HPV. If the test result is “positive,” the participant is confirmed to be HPV infected; If the result is “negative,” the participant judged to be non-HPV infected. This process ensures accurate and reliable diagnostics. Previous studies have validated the reliability of the self-reported HPV infection diagnosis in the questionnaire (20, 21).

### 2.3 Other covariates

Building on knowledge from previous research and clinical practice (22, 23), we factored in numerous covariates that could potentially mediate the association between magnesium intake and HPV infection. Continuity covariates include age, poverty income ratio (PIR), dietary intake of vitamins K, A, and C, and folic acid and beta-carotene intake. The categorical variables included racial/ethnic background, level of education, marital status, smoking history (i.e., cumulative smoking of 100 or more cigarettes in a lifetime), and drinking behavior (i.e., cumulative drinking of 12 or more drinks in a year). Specific information on all of these covariates is available via the authoritative NHANES online platform. Measurements of different variables detailed process in NHANES database’s official website^1^ are described.

### 2.4 Analytical statistics

We conducted a descriptive statistical analysis, presenting continuous variables as mean ± standard error (SE) and categorical variables as frequency (percentage), with mean, median, and standard error (SE) calculated for all variables. Differences between HPV-infected and non-infected groups were assessed using ANOVA for continuous variables and Chi-square tests for categorical frequencies. To address the complex sampling design of NHANES, weighted univariate analyses incorporating the first-day dietary recall sampling weights (WTDRD1/7) were implemented to minimize sampling bias and enhance national representativeness. Multivariate logistic regression models were constructed to examine magnesium intake’s association with HPV status (categorized dichotomously), with three progressively adjusted models: Model 1 (unadjusted), Model 2 (adjusted for age, race, and education), and Model 3 (fully adjusted for all covariates). Subgroup analyses using fully adjusted models and threshold effect evaluations via Smooth Curve Fitting were performed to identify population-specific associations and inflection points, quantified through adjusted odds ratios (ORs) with 95% confidence intervals (CIs). Sensitivity analyses addressed dietary variability by averaging magnesium intake from two 24-h recalls. To control the risk of Type I errors caused by multiple comparisons, this study adopted a stratified Bonferroni-corrected strategy. For the interaction tests between subgroups (a total of 9 subgroup variables), the post-correction significance threshold *α*’ = 0.05/9 ≈ 0.00556 was set, with statistical significance defined only when *p* < 0.00556. Within subgroups, the threshold was dynamically adjusted based on the number of strata, α” = 0.05/k (where k is the number of strata within a subgroup; for example, if age is divided into 4 strata, α = 0.05/4 = 0.0125). Although Bonferroni-correcting may increase the risk of Type II errors, we prioritize strict control over false-positive results. All analyses utilized R 4.4.3 and EmpowerStats, with effects rounded to three decimal places to mitigate rounding bias, maintaining a significance threshold of *p* < 0.05 for clinically meaningful outcomes.

## 3 Results

### 3.1 Basic demographics

Our study included 7,246 individuals aged 18–59, with 3,228 (44.55%) diagnosed with HPV. Table 1 summarizes the baseline demographics of these participants, stratified by their HPV infection status. In particular, the mean age of HPV-positive women (38.32 years) compared to HPV-negative women (39.76 years) demonstrated a highly statistically significant difference (*p* < 0.001). At the same time, HPV-infected and non-HPV-infected groups showed statistically significant differences (*p* < 0.05) in several aspects, including household income, race, marital status, education level, and vitamin A, vitamin C, vitamin K, and beta-carotene intake, smoking habits, and alcohol habits. Specifically, lower household income, lower education levels, lower intakes of vitamins A, C, K, and beta-carotene, and non-Hispanic black women who are divorced, with histories of smoking and alcohol use, all were correlated with an elevated risk for HPV infection. The mean intake of magnesium in HPV-infected persons (253.82 mg, standard deviation 4.36) was significantly lower than that in non-infected persons (278.06 mg, standard deviation 3.61), and the difference was statistically noteworthy, with a *p*-value < 0.05. Besides, when the participants were categorized into various groups according to the quartile of dietary magnesium intake, the differences in magnesium intake among the groups remained statistically significant (*p* < 0.05).

**Table 1:** Weighted baseline demographics of the study population.

Characteristics	HPV infection		*P*-value
	Yes (*n* = 3,228)	No (*n* = 4,018)	
Age, Mean (SE), years	38.32(0.30)	39.76(0.25)	**<0.001**
Poverty-to-income ratio, Mean (SE)	2.27 (0.05)	2.79(0.05)	**<0.001**
Race, *n* (%)			**<0.001**
Mexican American	504 (7.66%)	760 (8.65%)	
Other Hispanic	279 (5.62%)	339 (4.59%)	
Non-Hispanic White	1,326 (62.66%)	1935 (71.59%)	
Non-Hispanic Black	907 (18.26%)	598 (7.96%)	
Other Race	212 (5.80%)	386 (6.85%)	
Education attainment, n (%)			**0.005**
Below high school	694 (15.68%)	764 (12,65%)	
High school and above	2,534 (84.32%)	3,254 (87.35%)	
Marital status, n (%)			**<0.001**
Married	1,289 (42.14%)	2,580 (67.03%)	
Divorce	500 (16.90%)	324 (7.59%)	
Separated	155 (3.80%)	113 (1.96%)	
Never married	1,284 (37.15%)	1,001 (23.421%)	
Consumed at least 100 cigarettes in a lifetime, n (%)			**<0.001**
Yes	1,513 (49.69%)	1,341 (36.35%)	
No	1715 (50.31%)	2,677 (63.65%)	
Consumed at least 12 alcoholic drinks annually, n (%)			**<0.001**
Yes	2,335 (77.73%)	2,623 (71.80%)	
No	893 (22.27%)	1,395 (28.20%)	
Dietary vitamin K, Mean (SE), mg	6.84(0.17)	7.46(0.15)	**<0.001**
Dietary vitamin A, Mean (SE), mcg	574.07(19.76)	615.19(16.26)	**0.033**
Dietary vitamin C, Mean (SE), mg	80.42(1.96)	86.46(1.72)	**<0.001**
Folic acid, Mean(SE),mcg	177.41(5.24)	177.37(4.37)	0.746
Beta-carotene, Mean (SE),mcg	2022.44(115.99)	2276.50(96.10)	**0.023**
Magnesium intake, Mean (SE), mg	253.82(4.36)	278.06(3.61)	**<0.001**
Magnesium intake, n (%)			**<0.001**
Q1 (0–173)	942 (27.82%)	846 (19.20%)	
Q2 (174–244)	846 (26.22%)	981 (23.28%)	
Q3 (245–332)	749 (22.600%)	1,066 (27.69%)	
Q4 (333–1,500)	691 (23.36%)	1,125 (29.83%)	

Mean (S.E) stands for the arithmetic mean and its standard error, while *n* (%) is used to show the value and its percentage. Statistical significance results will be presented in [bold].

### 3.2 Correlation between magnesium intake and risk of HPV infection

**Figure 2:**
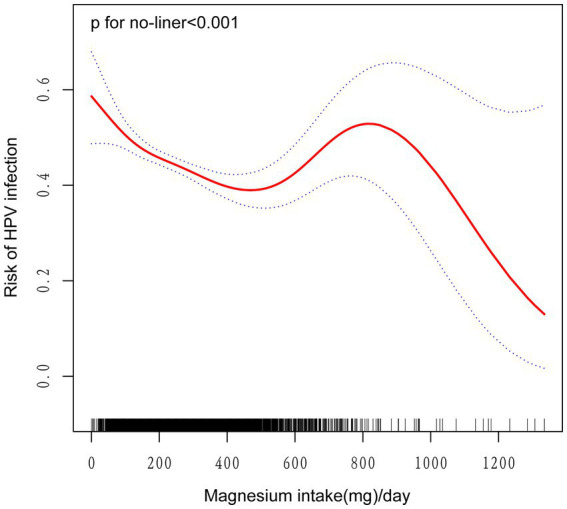
Correlation between Magnesium intake and the risk of HPV infection. The red solid line depicts the fitted smooth curve relating the variables, and the blue dashed line denotes the 95% confidence interval for this fit. Adjusted for age, race, poverty-to-income ratio, education level, marital status, Smoking status, drinking status, Intake of vitamins A, C, K, folic acid, and *β* -carotene.

To rigorously evaluate the potential association between magnesium consumption and HPV infection, we carefully designed three analysis models and integrated the key analysis results in Table 2. The statistical analysis demonstrated a significant negative correlation between magnesium intake and the risk of HPV infection. In detail, the unadjusted model revealed an odds ratio (OR) of 0.999 with a 95% confidence interval (CI) ranging from 0.998 to 0.999 (*p* < 0.0001). Similarly, the partially adjusted model(adjusted age, race, and education level) showed an OR of 0.999 (95% CI: 0.999–1.000, *p* = 0.018). The fully adjusted model also indicated an OR of 0.999 (95% CI: 0.999–1.000, *p* = 0.052), consistently suggesting an inverse relationship between magnesium consumption and HPV infection risk. When magnesium intake was categorized into quartiles, transforming it from a continuous to a categorical variable, the analysis results showed that in all three models, the HPV infection rate of individuals in the third (Q3) and fourth (Q4) quartile array showed a statistically significant decrease compared with the first quartile array (Q1) (*p* < 0.05). After comprehensively accounting for and adjusting for all potential confounding factors in Model 3, we discovered a notable decrease of 29.7% in the likelihood of HPV infection as magnesium intake transitioned from the lowest quartile to the highest quartile. The size of this effect was expressed as an OR of 0.703, with 95% CI ranging from 0.554 to 0.894, and the statistical significance level of this result was *p* = 0.005. In addition, the trend relationship between magnesium intake and the risk of HPV infection was highly statistically significant in all of the models constructed. Specifically, both Model 1 and Model 2 yielded trend test *p*-values of less than 0.001, which strongly indicates a very significant dose–response relationship between the two. Similarly, in model 3, the *p*-value of the trend test reaches 0.001, which also fully verifies the statistical significance of the trend. In addition, we employed smooth curve fitting methodologies to delve deeper into the potential relationship between magnesium intake and HPV risk. In doing so, we carefully adjusted for A range of covariates that included an individual’s age, family economic status, ethnic affiliation, marital status, education level, **s**moking habits, alcohol habits, and intake of vitamins A, C, K, folate, and beta-carotene, among other nutrients. The study results illustrate an L-shaped pattern of links between increased magnesium intake and decreased risk of HPV infection, as demonstrated in Figure 2. Through a detailed threshold effect analysis (the results are listed in Table 3), we observed a saturation effect at a magnesium intake of 401 units. Specifically, when magnesium intake was below 401 units, an increment of one unit in magnesium intake was linked to a 0.2% decrement in the risk of HPV infection. This effect was represented by an OR of 0.998, with a 95% CI precisely defined between 0.998 and 0.999, and the relationship observed was notably significant, yielding a *p*-value below 0.001. However, further increases in magnesium intake beyond this inflection point limit show not show a statistically significant link with the risk of HPV infection. Specifically, the effect size of magnesium intake and HPV risk at this stage was quantified by an OR of 1.001, with a 95% CI of 1.000 to 1.002, and a *p*-value of 0.155 for statistical tests, indicating that the association did not achieve statistical significance.

**Table 2:** Correlation between Magnesium intake and the risk of HPV infection.

Variable	Model 1		Model 2		Model 3	
	OR (95% CI)	*P* value	OR (95% CI)	*P* value	OR (95% CI)	*P* value
Magnesium intake (mg)	0.999 (0.998, 0.999)	**<0.001**	0.999 (0.999, 1.000)	**0.018**	0.999 (0.999, 1.000)	0.052
Magnesium intake quartile						
Q1 (0–173)	Ref.	Ref.	Ref.	Ref.	Ref.	Ref.
Q2 (174–244)	0.777 (0.650, 0.929)	**0.007**	0.862 (0.719, 1.034)	0.113	0.872 (0.718, 1.058)	0.170
Q3 (245–332)	0.563 (0.466, 0.681)	**<0.001**	0.664 (0.548, 0.805)	**<0.001**	0.671 (0.539, 0.834)	**<0.001**
Q4 (333–1,500)	0.540 (0.444, 0.658)	**<0.001**	0.688 (0.561, 0.843)	**<0.001**	0.703 (0.554, 0.894)	**0.005**
P for trend		**<0.001**		**<0.001**		**0.001**

Statistically significant values are denoted in the [bold] typeface. Model 1: This model excluded any accommodations for covariates. Model 2: This model included adjustments for age, racial, and education level factors. Model 3: Adjustments in this model conclude age, race, poverty-to-income ratio, marital status, education level, drinking status, Smoking status, Intake of vitamins A, C, K, folic acid, and β -carotene.

**Table 3:** Analysis of the threshold effect between magnesium intake and the risk of HPV infection.

Variable	Adjusted OR (95% CI)	*P* value
Fitting by the 2-piecewise linear model		
Inflection point	401	
Magnesium intake<401	0.998 (0.998, 0.999)	**<0.001**
Magnesium intake> = 401	1.001 (1.000, 1.002)	0.155
P for Log-likelihood ratio	**<0.001**	

Adjustments were made for age, poverty-to-income ratio, race, marital status, education, smoking status, alcohol consumption, and intake of vitamins A, C, K, folic acid, and β-carotene. Statistically significant values are denoted in the [bold] typeface.

### 3.3 Subgroup analysis

To further elucidate the connection between magnesium intake and HPV infection, we performed a thorough subgroup analysis (see Table 4 for the results). These subgroup analyses were classified and stratified by age, education level, marital status, vitamin A, vitamin K, vitamin C, beta-carotene intake, smoking history, and alcohol consumption to conduct a comprehensive analysis of the possible connection between magnesium intake and HPV risk among diverse subgroups of people. The study data revealed a notable interaction between vitamin A intake and magnesium intake in relation to the risk of HPV infection (p interaction = 0.033). Specifically, for each additional unit of vitamin A intake in the range of 243 to 453 μg/day, the risk of HPV infection was reduced by 0.2% (OR = 0.998, 95% CI: 0.997 to 0.999, *p* = 0.001). Evaluation of the interaction effects in all other subgroups showed that the association between magnesium intake and HPV infection risk was not statistically significant in all subgroups examined (all p interaction values >0.05), suggesting that differences between subgroups did not significantly affect this negative association. We used Bonferroni to correct for subgroups (Supplementary Table S2). After correction, there was no significant interaction test in the subgroups, indicating that the protective effect of magnesium was consistent in all subgroups. But in the subgroup with vitamin A intake of 243–453 μg/d (corrected *p* = 0.004 < 0.0125), dietary magnesium was still negatively correlated with HPV infection risk, and remained robust after Bonferroni correction.

**Table 4:** Weighted subgroup and interactive analyses of the correlation between magnesium intake and HPV infection.

Subgroup	OR (95%CI)	*P*-value	P-interaction
Age, years			0.169
20–28	0.999 (0.997, 1.000)	**0.010**	
29–38	1.000 (0.999, 1.001)	0.872	
39–48	1.000 (0.998, 1.001)	0.548	
49–59	0.999 (0.998, 1.000)	0.027	
Education attainment			0.915
Below high school	0.999 (0.998, 1.000)	0.244	
High school and above	0.999 (0.998, 1.000)	**0.035**	
Marital status			0.265
Married	0.999 (0.998, 1.000)	0.058	
Divorce	1.000 (0.999, 1.002)	0.741	
Separated	0.998 (0.995, 1.001)	0.117	
Never married	0.999 (0.999, 1.000)	0.135	
Dietary vitamin A, mcg			**0.033**
0–242	0.998 (0.996, 1.000)	0.060	
243–453	0.998 (0.997, 0.999)	**0.001**	
454–765	1.000 (0.998, 1.001)	0.650	
766–20,313	1.000 (0.999, 1.001)	0.986	
Dietary vitamin K, mg			0.095
0–30.8	0.998 (0.996, 0.999)	**0.041**	
30.9–55.2	0.999 (0.998, 1.000)	0.097	
55.3–108.5	1.000 (0.998, 1.001)	0.525	
108.6–6521.7	1.000 (0.999, 1.001)	0.995	
Dietary vitamin C, mg			0.092
0–20.3	0.998(0.997, 0.999)	**0.008**	
20.4–53.4	0.999(0.998, 1.000)	0.175	
53.6–117	1.000(0.999, 1.001)	0.719	
117.2–1077.6	1.000(0.999, 1.001)	0.703	
Beta-carotene, mcg			0.965
0–268	0.999 (0.998, 1.000)	0.181	
269–712	0.999 (0.998, 1.000)	0.205	
713–2,334	0.999 (0.998, 1.000)	0.232	
2,335–104,259	0.999 (0.998, 1.000)	0.278	
Consumed at least 100 cigarettes in a lifetime			0.697
Yes	0.999 (0.999, 1.000)	0.156	
No	0.999 (0.998, 1.000)	0.066	
Consumed at least 12 alcoholic drinks annually			0.440
Yes	0.999 (0.999, 1.000)	0.120	
No	0.999 (0.997, 1.000)	0.109	

To explore how the interaction between covariates and magnesium intake affects HPV infection rates, we performed a subgroup analysis. Logistic regression analysis was then used to assess the significance of these interactions, and the resulting P-value was used as a measure of statistical significance. Statistically significant values are denoted in the [bold] typeface.

### 3.4 Sensitivity analysis

Consistent with the results of the single-day dietary evaluation, the sensitivity analysis was used to confirm that the average dietary magnesium intake over 2 days and the results of HPV infection remained robust. It was consistently observed in both the unadjusted and adjusted models. For Model 3, the adjusted OR, 95%CI, and *p* value for the highest quartile (Q4) were 0.703 (0.554–0.894), 0.005 in the main analysis vs.0.751(0.592–0.953), 0.023 in the sensitivity analysis. Full results are provided in Supplementary Table S1. Overall, the sensitivity analysis confirmed the stability and reliability of the results obtained through the single-day dietary review logistic regression analysis.

## 4 Discussion

This study marks the inaugural systematic investigation into the potential link between dietary magnesium intake and the risk of human papillomavirus (HPV) infection. By independently employing multivariate logistic regression analysis, we have conducted a thorough assessment of the specific impact of magnesium intake on the risk of HPV infection. The findings revealed a notable inverse relationship between magnesium intake and the HPV infection risk, and this association remained consistent even after accounting for various other factors.

Despite the scarcity of studies exploring the connection between magnesium intake and HPV infection, existing evidence has consistently highlighted a notable relationship between serum magnesium concentrations and disorders of the female reproductive system, particularly pelvic inflammatory disease (PID). The research suggests that increasing magnesium intake through dietary means may have a favorable effect on decreasing the likelihood of PID by modulating immune responses and mitigating oxidative stress indicators, including malondialdehyde (MDA) levels (24). A crucial role has been identified for magnesium deficiency in elevating the risks associated with various pregnancy complications, including gestational diabetes, preeclampsia, preterm birth, and low birth weight in newborns (25–27). Additionally, a study of 2,304 Chinese women found a link between higher magnesium intake from diet and lower cervical cancer risk. Increasing magnesium consumption may help reduce cervical cancer prevalence and potentially slow its progression (28). Meanwhile, emerging evidence highlights a link between magnesium (Mg^2+^) and viral infections. For instance, XMEN disease, a rare X-linked immunodeficiency, is characterized by intracellular Mg^2+^ deficiency, resulting in impaired control of Epstein–Barr virus (EBV) and an increased risk of EBV-driven malignancies (29). Additionally, epidemiological data suggest that regions with lower magnesium levels exhibit a higher cumulative incidence of COVID-19, potentially due to Mg^2+^'s immunomodulatory effects and its synergistic interaction with vitamin D3 (30). The previous research results provide important theoretical support and background information for our current research. On the basis of this evidence, we not only conclusively established the close relationship between the consumption of magnesium in the diet and the occurrence of HPV infection but also innovatively depicted the L-shaped relationship between the two and accurately identified the inflection point of the curve. This breakthrough discovery opens up a new research direction for the application of dietary magnesium in the field of HPV infection prevention.

While the precise mechanisms underlying the inverse relationship between magnesium intake and HPV infection remain incompletely elucidated, this study proposes that adequate magnesium consumption may potentially inhibit HPV invasion and subsequent viral replication through two primary pathways: enhancement of immune defense mechanisms and improvement of antioxidant capacity. Firstly, magnesium is essential to ensure the integrity of epithelial barrier function. Mg2 + and Ca2 + play a key role in stabilizing biofilms by neutralizing and cross-linking the charge of lipid carboxylation and phosphorylation of head groups, thus effectively defending against the adsorption of viruses (such as HPV). Inadequate magnesium intake may weaken the stability of cell membranes and increase the risk of HPV infection (31). Furthermore, magnesium may influence viral replication by regulating the DNA-binding ability of the HPV E2 protein. Studies indicate that E2 requires Mg^2+^ for stable binding to the viral origin of replication (ori). In magnesium-deficient conditions, E2’s DNA-binding affinity decreases, potentially disrupting viral replication control (32, 33). An alternative mechanism may involve magnesium’s role in suppressing HPV replication through upregulation of interferon-*γ* (IFN-γ) production. This enhanced IFN-γ signaling not only potentiates the host’s innate immune response but also confers cellular resistance to intracellular viral infection (34–37). Notably, IFN-*γ* suppresses HPV E6/E7 oncogene expression through multiple pathways. First, IFN-γ activates the JAK–STAT signaling cascade, leading to STAT protein nuclear translocation and binding to interferon-stimulated response elements (ISREs) in target gene promoters. This induces antiviral and immunoregulatory proteins, some of which interfere with the HPV transcriptional machinery, disrupting E6/E7 transcription initiation and reducing oncogene expression (3, 4). Second, IFN-γ upregulates microRNAs (miR-34a) that target the 3’UTR of E6/E7 mRNAs, promoting their degradation or translational inhibition, further downregulating oncoprotein levels (38).

Furthermore, magnesium ions (Mg2+) serve as essential cofactors in multiple fundamental DNA repair pathways, including three major mechanisms: nucleotide excision repair (NER), base excision repair (BER), and mismatch repair (MMR) (10). Inadequate Mg2 + intake may impair the ability of host cells to carry out these DNA repairs, which in turn may contribute to the worsening process of HPV infection. Moreover, in biological metabolic processes, magnesium ions (Mg2+) function as crucial cofactors for adenine nucleotides, exerting central regulatory control over multiple rate-limiting enzymes in the glycolytic pathway, including hexokinase and phosphofructokinase. This essential role establishes Mg2 + as an indispensable component in cellular glucose metabolism (39). Mg2 + plays a central role in ensuring adequate energy for cell activity. A lack of magnesium ions will disrupt the normal process of cell metabolism and may prevent normal cells from obtaining energy and the necessary material basis, thereby affecting the body’s immune function, so that the risk of HPV infection increases. During antigen-specific immune responses, reduced extracellular magnesium concentration triggers a cascade of cellular events, including the activation of voltage-gated L-type calcium channels and N-methyl-D-aspartate (NMDA) receptors. This receptor-mediated signaling pathway ultimately results in elevated intracellular calcium ion concentrations. This molecular cascade subsequently triggers the biosynthesis and release of substance P, a neuropeptide that activates the nuclear factor-kappa B (NF-κB) signaling pathway. The activated NF-κB pathway then upregulates the transcription and secretion of pro-inflammatory cytokines, particularly tumor necrosis factor-alpha (TNF-*α*) and interleukin-6 (IL-6) (40). IL-6 enables HPV-infected cells to evade T cell-mediated immune responses, and at high concentrations, it may play a significant role in the persistence of HR-HPV infection and disease progression (41). Additionally, Mg2 + enhances immune synapse formation by facilitating the transition of the co-stimulatory molecule LFA-1 on CD8 + T cells into its active conformation, thereby augmenting antigen-specific cytotoxicity (42, 43). In addition, magnesium can further strengthen the antiviral immune response by modulating T-cell receptor (TCR) signaling, which in turn enhances the ability of CD8^+^ T cells to recognize HPV E2/E6/E7 antigens (44, 45). The impact of magnesium on HPV oncoproteins E6/E7 is primarily mediated through indirect pathways. A low-magnesium environment may activate the DNA damage response by exacerbating HPV-induced oxidative stress, thereby enhancing E2 gene activity to indirectly suppress E6/E7 expression, thus preventing replicative immortality (46, 47). Consequently, adequate magnesium ion (Mg2+) levels are crucial for optimizing T cell-mediated immune responses against HPV and other viral infections. Conversely, magnesium deficiency impairs T-cell functionality, compromising their capacity to eliminate HPV-infected cells.

Besides, accumulating evidence suggests that magnesium deficiency may not only initiate a self-perpetuating cycle of chronic low-grade inflammation but also potentiate HPV-induced inflammatory stress responses (40). Inflammatory cascades trigger the secretion of multiple pro-inflammatory mediators, including interleukin-1 (IL-1), interleukin-6 (IL-6), and tumor necrosis factor-alpha (TNF-*α*). These pro-inflammatory cytokines initiate protein kinase-dependent signaling cascades that subsequently induce reactive oxygen species (ROS) generation (48). This oxidative stress-mediated mechanism can cause genomic instability through DNA damage, representing a critical molecular pathway in HPV-associated malignant transformation (49). Oxidative stress (OS) causes host cell DNA and protein damage while also activating the DNA damage response pathway, particularly during HPV infection (50). During double-strand break events, TOPBP1 is recruited to damage sites where it coordinates with other repair proteins to form a response complex. Notably, TOPBP1 potentiates HPV E2 protein activity, enhancing both viral transcription and genome replication (51). Oxidative stress (OS) not only induces DNA and protein damage in host cells but also, particularly during HPV expression, enhances the activity of viral components E1 and E2 in the DNA replication complex through activation of the DNA damage response pathway. This mechanism enables viral replication to persist despite DNA damage, ultimately leading to increased viral amplification and genomic rearrangement (50). In the context of primary HPV infection or high infection rates, magnesium enhances immunologic synapse efficacy by activating the LFA-1 protein on T cells and modulates inflammatory factors (IL-6), indirectly supporting antiviral immunity. However, direct evidence of magnesium inhibiting viral replication remains lacking, with its primary value lying in maintaining immune homeostasis to reduce infection risk. All these indicate that maintaining appropriate magnesium intake and magnesium balance in the body is extremely important for the body to effectively resist HPV infection.

Notably, for immunocompromised patients, appropriate magnesium supplementation may indirectly improve immune function by enhancing T cell activity (LFA-1-mediated immunologic synapse formation) and supporting energy metabolism (ATP production, protein synthesis). However, magnesium over-supplementation should be avoided in individuals with normal renal function. In dialysis patients, magnesium metabolism disorders (hyper−/hypomagnesemia) due to impaired renal excretion necessitate regular serum magnesium monitoring, with magnesium homeostasis maintained via dialysis fluid adjustment and personalized dietary management (52). For cervical cancer patients, degradation products of magnesium alloy biomaterials (Mg^2+^, OH^−^, H_2_) inhibit SiHa cell proliferation and induce apoptosis by accumulating free radicals in the tumor microenvironment and triggering G0/G1 cell cycle arrest. However, these antitumor effects do not directly target HPV itself. At the carcinogenic stage, viral DNA has already integrated into the host genome, rendering viral clearance insufficient to reverse cancer progression (53, 54).

Simultaneously, by examining the L-shaped curve and threshold effect, we determined the threshold for magnesium intake to be 401 mg, marking a pivotal turning point. In other words, once magnesium intake exceeds a certain threshold, continuing to increase magnesium intake will not further reduce HPV infection rates. The plausible physiological mechanism for this observation may be attributed to an inverse association between magnesium ingestion and estrogen levels. Existing studies have indicated a distinct link between decreased estrogen concentrations and higher magnesium levels in women (55–57). Estrogen is essential for the regulation of immune response, and it affects many aspects of cytokine production, cellular chemotactic behavior, and phagocytosis (58–60). Therefore, the decrease in estrogen levels in a high-magnesium environment may weaken the body’s immune response and reduce its defense against HPV. This series of physiological chain reactions eventually leads to a tipping point, when magnesium intake exceeds a certain limit, its protective effect against HPV infection will plateau and no longer increase significantly with the increase of magnesium intake. Significantly, this threshold exceeds the NIH’s recommended daily magnesium intake for women (310–320 mg/day) by 25–30%, yet remains below established safety limits (61, 62). Thus, in clinical use, a moderate increase in magnesium intake to this threshold level under physician supervision may provide additional protection.

Further, the subgroup analysis of this study revealed that when vitamin A intake was 243–453 μg/d, dietary magnesium exhibited a significantly inverse association with HPV infection risk (OR = 0.998, 95% CI: 0.997–0.999, *p* = 0.001), with a notable interaction initially observed (P-interaction = 0.033). After Bonferroni correction, the association remained significant within the subgroup (0.004 < 0.0125), while the interaction lost significance (0.297 < 0.00556), suggesting magnesium’s protective effect might be consistent across vitamin A intake subgroups, though cautious interpretation is warranted. Despite the interaction not surviving multiple testing correction, the core finding—magnesium’s protective effect in the 243–453 μg/d vitamin A subgroup—remained robust post-correction. Mechanistically, vitamin A may activate the retinoic acid (RA)-retinoic acid receptor *α* (RARα) axis to enhance interferon-*γ* (IFN-γ) secretion in cervical mucosal epithelial cells, thereby strengthening antiviral responses and mucosal barrier function (63–65), while magnesium is shown to upregulate IFN-γ production to inhibit HPV replication (34), indicating potential synergistic immunomodulation. Additionally, vitamin A protects biomolecules from ROS-induced oxidative damage (66), and magnesium mitigates mitochondrial ROS production while enhancing antioxidant defenses (67), collectively suppressing HPV-induced DNA damage and viral integration. Notably, vitamin A within this dose range might achieve the RARα signaling activation threshold, optimizing magnesium’s immunomodulatory effects, whereas exceeding this range could introduce nutritional antagonism. Although the OR approaches 1, its high significance (*p* = 0.001) implies a subtle yet consistent population-level biological effect. Future studies should validate HPV infection rate dynamics and dose–response relationships in clinical cohorts.

Several significant strengths stand out in our study, the most critical of which is our pioneering use of the NHANES database to analyze the interplay between magnesium consumption and HPV infection. Each year, the NHANES database selects a meticulously curated group of around 5,000 participants from various locations throughout the United States who represent a wide range of socioeconomic status, age groups, and ethnic backgrounds, demonstrating a high degree of population diversity. By integrating and analyzing valuable data accumulated over nearly 14 years, we successfully included a large sample of 7,246 women, which significantly improved the dependability and precision of our statistical discoveries. In addition, our study also deepened the level of subgroup analysis through detailed analysis of the interplay between magnesium intake levels and the presence of HPV infection in different subgroups of people to further enhance the reliability and depth of the study conclusions.

Our investigation faced several key limitations, the first being the use of a cross-sectional study design, which hampered our ability to accurately establish the causal relationship between magnesium intake and HPV infection. At the same time, focusing only on a sample of the US population may also limit the applicability of our findings to a broader geographic and demographic context. Because this cohort exhibited significant diversity in dietary patterns, HPV infection status, and genetic background, our findings may face limitations in applicability to other demographic groups. Furthermore, although we have corrected multiple covariates such as population, diet, and lifestyle, due to the limitations of the current data, we cannot completely rule out other potential confounding factors that may have an impact on magnesium intake and HPV infection. In addition, although the data collection work of NHANES is based on standardized personal interviews and physical examination procedures, the dietary recall interview itself is associated with the danger of memory bias. Aspects like sharpness of memory, level of cognitive function, cultural background information differences, and personal eating habits may weaken the accuracy of dietary data obtained through this interview. To gain a more comprehensive insight into the causal relationship between magnesium consumption and HPV infection, future research should prioritize adopting either a longitudinal or cohort study design. These methods will allow us to obtain more detailed time series data and deeper causal analysis, thereby promoting our cognitive development in this area of science.

## 5 Conclusion

In short, our study found an L-type relationship between magnesium intake and the risk of HPV infection, suggesting that increasing magnesium intake before 401 mg can effectively enhance protection against HPV infection. At the same time, our study also found that when vitamin A intake is maintained ranging from 243 to 453 MCG/day, there may be a synergistic effect with magnesium intake to help reduce HPV infection, which opens up new avenues for prevention and treatment of HPV infection through dietary management.

## Data Availability

The raw data supporting the conclusions of this article will be made available by the authors, without undue reservation.

## Footnotes

^1^ https://www.cdc.gov/nchs/nhanes/ContinuousNhanes/Default.aspx
