# Supplementary material for: L-type association between magnesium intake and human papillomavirus infection in US adult women: based on NHANES 2003–2016 data
Source: Front Nutr. 2025 May 30;12:1594489. doi: 10.3389/fnut.2025.1594489 (PMC12163323; doi:10.3389/fnut.2025.1594489)
Supplement: Supplementary file 1 [file Table_1.docx]

**Table S1: Sensitivity Analysis of Magnesium Intake and HPV Infection: Single vs. Two 24-Hour Dietary Recalls**

|  | **Model 1**  **OR (95% CI)P value** | | **Model 2**  **OR (95% CI)P value** | | **Model 3**  **OR (95% CI)P value** | |
| --- | --- | --- | --- | --- | --- | --- |
|  | **Single recall** | **Two**  **recalls** | **Single recall** | **Two**  **recalls** | **Single recall** | **Two**  **recalls** |
| **Magnesium intake (mg)** | 0.999 (0.998, 0.999)**<0.001** | 0.998 (0.998, 0.999)**<0.001** | 0.999 (0.999, 1.000)**0.018** | 0.999 (0.998, 1.000)**0.036** | 0.999 (0.999, 1.000)0.052 | 0.999(0.998, 1.000)0.119 |
| **Magnesium intake**  **quartile** |  |  |  |  |  |  |
| Q1 | Ref. | Ref. | Ref. | Ref. | Ref. | Ref. |
| Q2 | 0.777 (0.650, 0.929)**0.007** | 0.732 (0.593, 0.903)**0.005** | 0.862 (0.719, 1.034)0.113 | 0.828 (0.662, 1.036)0.103 | 0.872 (0.718, 1.058)0.170 | 0.844(0.665, 1.071)0.167 |
| Q3 | 0.563 (0.466, 0.681)**<0.001** | 0.642 (0.536, 0.767)**<0.001** | 0.664 (0.548, 0.805)**<0.001** | 0.787 (0.654, 0.948)**0.0134** | 0.671 (0.539, 0.834)**<0.001** | 0.844(0.692, 1.029)0.098 |
| Q4 | 0.540 (0.444, 0.658)**<0.001** | 0.549 (0.449, 0.671)**<0.001** | 0.688 (0.561, 0.843)**<0.001** | 0.713 (0.580, 0.877)**0.002** | 0.703 (0.554, 0.894)**0.005** | 0.751(0.592, 0.953)**0.023** |

Note:

Statistically significant values are denoted in the [bold] typeface.

Model 1: This model excluded any accommodations for covariates.

Model 2: This model included adjustments for age, racial, and education level factors.

Model 3: Adjustments in this model conclude age, race, poverty-to-income ratio, marital status, education level, drinking status, Smoking status, Intake of vitamins A, C, K, folic acid, and β -carotene.

The single dietary data is from Day1, and the two dietary data are the mean of Day1 and Day2.

**Table S2: Subgroup Analysis of Magnesium Intake and HPV Infection Risk with Bonferroni Adjustment: NHANES 2003-2016**

| **Subgroup** | **Within-p** | **Within-p-adj** | **Interaction-p** | **Interaction-p-adj** |
| --- | --- | --- | --- | --- |
| **Age, years** |  |  | 0.169 | 1.000 |
| 20 - 28 | 0.010 | 0.040 |  |  |
| 29 - 38 | 0.872 | 1.000 |  |  |
| 39 - 48 | 0.548 | 1.000 |  |  |
| 49 - 59 | 0.027 | 0.108 |  |  |
| **Education attainment** |  |  | 0.915 | 1.000 |
| Below high school | 0.244 | 0.488 |  |  |
| High school and above | 0.035 | 0.070 |  |  |
| **Marital status** |  |  | 0.265 | 1.000 |
| Married | 0.058 | 0.232 |  |  |
| Divorce | 0.741 | 1.000 |  |  |
| Separated | 0.117 | 0.468 |  |  |
| Never married | 0.135 | 0.540 |  |  |
| **Dietary vitamin A, mcg** |  |  | 0.033 | 0.297 |
| 0 - 242 | 0.060 | 0.24 |  |  |
| 243 - 453 | 0.001 | **0.004** |  |  |
| 454 - 765 | 0.650 | 1.000 |  |  |
| 766 - 20313 | 0.986 | 1.000 |  |  |
| **Dietary vitamin K, mg** |  |  | 0.095 | 0.855 |
| 0 - 30.8 | 0.041 | 0.164 |  |  |
| 30.9 - 55.2 | 0.097 | 0.388 |  |  |
| 55.3 - 108.5 | 0.525 | 1.000 |  |  |
| 108.6 - 6521.7 | 0.724 | 1.000 |  |  |
| **Dietary vitamin C, mg** |  |  | 0.092 | 0.828 |
| 0 - 20.3 | 0.008 | 0.032 |  |  |
| 20.4 - 53.4 | 0.175 | 0.700 |  |  |
| 53.6 - 117 | 0.719 | 1.000 |  |  |
| 117.2 - 1077.6 | 0.703 | 1.000 |  |  |
| **Beta-carotene, mcg** |  |  | 0.965 | 1.000 |
| 0 - 268 | 0.181 | 0.724 |  |  |
| 269 - 712 | 0.205 | 0.820 |  |  |
| 713 - 2334 | 0.232 | 0.928 |  |  |
| 2335 - 104259 | 0.278 | 1.000 |  |  |
| **Consumed at least 100 cigarettes in a lifetime** |  |  | 0.697 | 1.000 |
| Yes | 0.156 | 0.312 |  |  |
| No | 0.066 | 0.132 |  |  |
| **Consumed at least 12 alcoholic drinks annually** |  |  | 0.440 | 1.000 |
| yes | 0.120 | 0.240 |  |  |
| no | 0.109 | 0.218 |  |  |

Note: Intra-group comparisons were corrected by stratified Bonferroni, and the threshold was dynamically calculated based on the number of stratifications (for example, age was divided into 4 layers, and the correction threshold α=0.0125);The interaction test was corrected by Bonferroni (total number of subgroups k=9, correction threshold α=0.00556) ;The P value were derived from the multivariate logistic regression model(Adjustments in this model conclude age, race, poverty-to-income ratio, marital status, education level, drinking status, Smoking status, Intake of vitamins A, C, K, folic acid, and β -carotene.)；Bold indicates that the p-value after correction is less than the corresponding threshold.
